# Relationship between p53 status and radiosensitivity in human tumour cell lines.

**DOI:** 10.1038/bjc.1996.101

**Published:** 1996-03

**Authors:** E. Siles, M. Villalobos, M. T. Valenzuela, M. I. Núñez, A. Gordon, T. J. McMillan, V. Pedraza, J. M. Ruiz de Almodóvar

**Affiliations:** Departamento de Radiologia y Medicina Fisica, Hospital Universitario, Facultad de Medicina, Granada, Spain.

## Abstract

**Images:**


British Journal of Cancer (1996) 73, 581-588
© 1996 Stockton Press All rights reserved 0007-0920/96 $12.00

### Relationship between p53 status and radiosensitivity in human tumour cell lines

E Siles[1], M Villalobos[1], MT Valenzuela[1], MI Núñez[1], A Gordon[2], TJ McMillan[2], V Pedraza[1] and JM Ruiz de Almodóvar[1]

[1]*Laboratorio de Investigaciones Médicas y Biología Tumoral, Departamento de Radiología y Medicina Física, Hospital Universitario, Facultad de Medicina, 18071 Granada, Spain;* [2]*Radiotherapy Research Unit, The Institute of Cancer Research, 15 Cotswold Road, Sutton, Surrey SM2 5NG, UK.*

**Summary** We examined the relationship between p53 levels before and after irradiation, radiation-induced cell cycle delays, apoptotic cell death and radiosensitivity in a panel of eight human tumour cell lines. The cell lines differed widely in their clonogenic survival after radiation, (surviving fraction at 2 Gy: SF2 = 0.18 − 0.82). Constitutive p53 protein levels varied from $2.2 \pm 0.4$ to $6.3 \pm 0.3$ optical density units (OD) per $10^6$ cells. p53 after irradiation (6 Gy) also varied between the cell lines, ranging from no induction to a 1.6-fold increase in p53 levels 4 h after treatment. p53 function was also assessed by $G_1$ cell cycle arrest after irradiation. The cellular response to radiation, measured as $G_0/G_1$ arrest, and the induction of apoptosis were in good agreement. However, a trace amount of DNA ladder formation was found in two cell lines lacking $G_1$ arrest. Overall cellular radiosensitivity correlated well with the level of radiation-induced $G_1$ arrest (correlation coefficient $r = 0.856$; $P = 0.0067$), with p53 constitutive levels ($r = 0.874$, $P = 0.0046$), and with p53 protein fold induction ($r = -0.882$, $P = 0.0038$). Our data suggest that (1) the constitutive p53 level, (2) $G_1$ arrest after irradiation, or (3) the p53 protein response to radiation may be good predictive tests for radiosensitivity in some cell types.

**Keywords:** radiation; cellular radiosensitivity; cell cycle delay; $G_1$ arrest; p53 response

It is generally accepted that ionising radiation kills eukaryotic cells by damaging the structure and function of genomic DNA. Much effort has consequently been focused on understanding how cells respond to DNA damage and restore the DNA sequence integrity and chromatin structure. Differences in the intrinsic radiosensitivity of human cells are now acknowledged, and the picture that emerges from the review of radiobiological data suggests that these differences may be related to: (a) the number of initial radiation-induced DNA double-strand breaks (dsbs) (Ruiz de Almodóvar *et al.*, 1994); (b) the number of unrejoined DNA dsbs (Wurm *et al.*, 1995); (c) the rate of rejoining of dsbs (Núñez *et al.*, 1995; Whitaker *et al.*, 1995) and (d) the fidelity of dsb rejoining (Powell and McMillan, 1994).

It has also been suggested that transient alterations in cell cycle progression in $G_1$ and $G_2$ phases after exposure to different DNA-damaging agents are important components of the cellular response to DNA damage (Kastan *et al.*, 1992; Canman *et al.*, 1994; Baker *et al.*, 1990). These alterations presumably permit optimal repair by delaying DNA replication ($G_1$ arrest) (Kastan *et al.*, 1992) and chromosome segregation ($G_2$ arrest) (Nagasawa *et al.*, 1994).

The function of p53 appears to form part of a negative regulator pathway of DNA synthesis leading to $G_1$ arrest after cellular exposure to DNA-damaging agents, since there is a close temporal association between the post-transcriptional increase in p53 protein levels and $G_1$ arrest after irradiation (Kastan *et al.*, 1991). In contrast, cells with mutant p53 genes or lacking p53 genes failed to show any increase in p53 protein after DNA damage; this correlates with a lack of $G_1$ arrest (Kastan *et al.*, 1991; Kuerbitz *et al.*, 1992), although these cells still show $G_2$ arrest. Stewart *et al.* (1995) recently suggested that the antiproliferative activity of p53 may be also involved in the $G_2/M$ restriction point.

Many studies have shown that most p53 mutations result in a non-functional protein that accumulates in tumour cells (Levine *et al.*, 1991; Hollstein *et al.*, 1991). It seems that p53 protein accumulation is a consequence of its stabilisation (Hall *et al.*, 1991; Schlichtholz *et al.*, 1992). Loss of p53 function as in mutant p53 was recently shown to increase the resistance to DNA-damaging agents in human tumour cell lines (McIlwrath *et al.*, 1994; Fan *et al.*, 1994). High constitutive levels of intracellular p53 levels may thus be related with radioresistance to ionising radiation; the relation between the cellular response to radiation-induced damage ($G_1$ block) and the triggering of apoptotic cell death may explain the differences in radiosensitivity.

To investigate this hypothesis we have used a panel of eight human tumour cell lines that differed widely in their clonogenic survival after radiation. We developed an immunoenzyme assay to measure constitutive p53 protein levels in whole human tumour cells attached to the monolayer. These data were compared with radiation-induced apoptosis, with the intrinsic cellular radiosensitivity values and with p53 functionality assessed through $G_1$ arrest and p53 induction.

### Materials and methods

### *Cell culture, radiation treatment and clonogenic assay*

Nine human tumour cell lines were studied, although one of them, HL60, was only used as a negative control in a set of experiments. Three different clones of the MCF-7 cell line originally established by Soule *et al.* (1973) were obtained from G Leclercq (Institut Jules Bordet, Brussels, Belgium), from C Sonnenschein (Tufts University, Boston, MA, USA) and from the American Type Culture Collection, named herein respectively MCF-7 BB, MCF-7 BUS (Ruiz de Almodóvar *et al.*, 1994) and MCF-7 GS. The EVSA-T human breast cancer line (Lippman *et al.*, 1976) was obtained from G Leclercq (Institut Jules Bordet, Brussels, Belgium). The clone of T47D human breast cancer cell line established by Keydar *et al.* (1979) and named T47D-B8 (Soto *et al.*, 1986) was obtained from C Sonnenschein. Cell line MDA-MB-231 was established by Cailleau *et al.* (1974). The RT-

Correspondence: JM Ruiz de Almodóvar
Received 12 June 1995; revised 19 October 1995; accepted 27 October 1995

112 human bladder carcinoma cell line (Masters *et al.*, 1986) was obtained from JRW Masters (The Institute of Urology, London, UK). Human medulloblastoma cell line D283MED (Friedman *et al.*, 1985) and myeloid leukaemia cell line HL60 (Collins *et al.*, 1977) were also used.

Cell cultures were grown in Dulbecco's modified Eagle medium (DMEM, Gibco) supplemented with 10% fetal calf serum (FCS) and incubated at 37°C in 95% air/5% carbon dioxide.

Cells in the exponential growth phase were irradiated using a cobalt-60 source at a dose rate of 1.67 Gy $min^{-1}$. For the flow cytometry experiments and for the p53 time course expression after cellular irradiation, a single 6 Gy dose was delivered. The radiation dose response for p53 induction was studied for a dose range from 2 to 8 Gy. Cellular survival after irradiation was assessed using acute-dose clonogenic assays performed in monolayer cultures as previously described (Ruiz de Almodóvar *et al.*, 1994; Núñez *et al.*, 1995). Survival data were fitted using the linear-quadratic model [$\ln SF = -(\alpha D + \beta D^2)$], with non-linear regression analysis. The values of survival fraction at 2 Gy (SF2) obtained from these fits are given in Table I.

*Flow cytometry*

At various times after $\gamma$-irradiation (6 Gy) ranging from 4 to 48 h, cell cycle analysis was done. After harvest, cells were suspended in full culture medium, centrifuged at 1200 r.p.m. for 3 min and stained with 1 ml Vindelov's solution containing $7.5 \times 10^{-5}$ M propidium iodide (PI) as described previously (Robinson, 1993). The cells were then incubated at 4°C for 10 min before running on an Ortho Cyteron absolute flow cytometer in which DNA content was used to distinguish each cell cycle phase. Quantification of cells in each cell cycle phase was done using the Ortho Cell Cycle program provided by the manufacturer. The proportion of cells in each cell cycle phase was expressed as a ratio of the percentage in unirradiated cells.

*p53 protein ELISA whole cell assay*

We used an immunoenzyme assay to measure the level of p53 protein in whole cells. Briefly, cells in exponential growth were harvested and counted, and appropriate numbers of cells were seeded in 24-well plates (Falcon) 24 h before the beginning of the assay in order to allow cells to attach to the culture flasks. Cells were then fixed with cold methanol acetone (1:1) for 10 min at 4°C, then rinsed with phosphate-buffered saline (PBS) to remove fixatives. Dry plates were stored until use. We added 150 $\mu$l of the polyclonal rabbit antiserum anti-p53 antibody CM-1 (Lander) which recognises conformational epitopes for both wild and mutant p53 proteins, diluted 1:1000 in 1% PBS with bovine albumin (BSA–PBS) and incubated the cells for 2 h at 4°C. The plates were then washed twice with 500 $\mu$l cold 1% BSA–PBS for 10 min at 4°C. The washing solution was removed and 150 $\mu$l of peroxidase-conjugated swine antiserum to the rabbit immunoglubulin (M701, Dako) diluted 1:1000 was added and the cells were incubated again for 2 h at 4°C. After a washing step as above, bound enzyme activity was detected with 200 $\mu$l of a 0.4 mg $ml^{-1}$ solution of ortho-phenylenediamine (OPD) peroxidase substrate (Sigma Fast, Sigma) according to the directions for use provided by the manufacturer. Aliquots from each well were transferred to wells in a 96-well microtitre plate, and results were monitored at 492 nm in an automatic plate reader (Titertek Multiskan plus, ICN Flow). This method allowed us to assess optical density values (corresponding to the p53 protein content in cells) ranging from $2.5 \times 10^4$ to $1.5 \times 10^5$ per well. The number of cells per well was checked again after the experiment was done.

*p53 Western blotting assay*

To measure p53 protein levels at different times after cell irradiation, cell extracts were prepared by lysing cells in 1% Nonidet P-40, 5% sodium deoxycholate and 0.1% sodium dodecyl sulphate in the presence of protease inhibitors. Cell extracts were stored at −80°C until use. Protein concentration was determined by the Bio-Rad protein assay, and 20 $\mu$g of protein was loaded onto an SDS-polyacrylamide gel. The gels were run at 150 V for 90 min in a Bio-Rad mini gel system. Proteins in the gel were transferred to a nitrocellulose membrane (100 V, 1 h) and then blocked for 1 h in 5% non-fat milk at room temperature. A polyclonal antibody to p53 (CM-1, Lander) was used for p53 protein determinations. Antibody reaction was revealed with chemiluminescence detection procedures according to the manufacturer's recommendations (ECL kit, Amersham).

*Assay for DNA fragmentation*

At the end of each incubation period after radiation, floating and adherent cells were centrifuged for 10 min at 900 *g* and washed with PBS. The pellet was resuspended in a lysis buffer (100 mM Tris-HCl pH 8, 10 mM EDTA, 10 mM sodium chloride, 2% SDS and 10 $\mu$l of a 10 mg $ml^{-1}$ solution of RNAase), and incubated at 37°C for 30 min. We then added 100 $\mu$g $ml^{-1}$ protein kinase and incubated the mixture at 37°C overnight. The DNA was extracted by phenol and chlorophorm–iso-amyl alcohol (24:1), precipitated overnight in −20°C ethanol containing sodium acetate at a final concentration of 0.3 M, centrifuged for 10 min, 4°C, at high speed (Microfuge, Beckman). The pellet was resuspended in Tris-EDTA buffer (0.1 M Tris-HCl, pH 8, 10 mM EDTA). The DNA samples (0.2 $\mu$g each) were electrophoretically separated on a 1% agarose gel containing ethidium bromide (0.5 $\mu$g $ml^{-1}$). DNA was visualised with an UV transilluminator, and the gels were photographed with a Polaroid camera.

**Table I** Cell lines, radiosensitivity, cell cycle arrest and constitutive levels of p53

| *Cell line* | *Origin*[a] | *SF2* | *$G_1$ arrest*[b] | *$G_2$ arrest*[c] | *p53*[d] | *Fold p53*[e] | *p53 status*[f] | *Apoptosis*[g] |
|---|---|---|---|---|---|---|---|---|
| MCF-7 BUS | 1 | 0.33±0.04 | 1.53 | 2.41 | 2.31±0.23 | 1.56 | wt | (+) |
| MCF-7 BB | 1 | 0.50±0.02 | 1.25 | 1.70 | 3.67±0.48 | 1.14 | mt | (–) |
| MCF-7 GS | 1 | 0.28±0.03 | 1.66 | 2.39 | 3.26±0.27 | 1.37 | wt | (+) |
| T47D-B8 | 1 | 0.55±0.01 | 1.33 | 1.37 | 3.64±0.31 | 1.16 | mt | (–) |
| EVSA-T | 1 | 0.65±0.03 | 1.08 | 2.45 | 3.38±0.45 | 0.90 | mt | (+/–) |
| MDA-MB-231 | 1 | 0.82±0.02 | 1.07 | 2.76 | 6.30±0.28 | 1.07 | mt | (–) |
| RT-112 | 2 | 0.68±0.02 | 1.00 | 2.20 | 4.65±0.29 | 1.01 | mt | (+/–) |
| D283MED | 3 | 0.18±0.01 | 1.42 | 1.86 | 2.08±0.24 | 1.38 | wt | (+) |
| HL60 | 4 | – | – | – | 0.00±0.00 | – | – | |

[a]1, Breast cancer cell line; 2, bladder carcinoma cell line; 3, medulloblastoma cell line; 4, myeloid leukaemia cell line. [b] Maximum ratio of cells in $G_1$ after irradiation compared to unirradiated cells. [c] Maximum ratio of cells in $G_2$ after irradiation compared to unirradiated cells. [d] p53 optical density units × $10^6$ cells measured in untreated cells: constitutive levels of p53; [e] p53-fold induction measured 4 h after cell irradiation. [f]wt, wild-type p53; mt, mutant-type p53. [g] (+), clear appearence of oligonucleosomal fragments; (+/–), trace amount of DNA ladder formation; (–), smear pattern.

### Results

*Clonogenic cell survival assay*

Table I shows the acute radiation dose–cell survival fraction at 2 Gy for all the cell lines assayed. Experiments were performed at least three times with each cell line, and pooled data were fitted to a linear-quadratic equation to obtain these estimates of the surviving fraction at 2 Gy. SF2 values ranged from 0.18 to 0.82. Cell line D283MED (medulloblastoma) was the most radiosensitive and MDA-MB-231 breast cancer cells were the most radioresistant.

*Radiation-induced cell cycle arrest*

In mammalian cells, exposure to radiation is known to induce both $G_1$ and $G_2$ arrests. After irradiation, time course experiments of cell cycle distribution were done. The growth arrest in $G_0/G_1$ and in $G_2/M$ as determined by PI staining

**Figure 1** Time course of $G_0/G_1$ ratio for irradiated (6 Gy) *vs* unirradiated cells, assessed by flow cytometry. Points represent means of at least three experiments; a minimum of 10 000 events were counted.

and DNA flow cytometry are shown in Figures 1 and 2. The maximum values obtained (Table I) allowed us to assess whether the p53 protein was functional. In spite of the limitations of PI staining, this method is widely used (Fan *et al.*, 1994; O'Connor *et al.*, 1993; Strasser *et al.*, 1994), and the patterns of radiation-induced cell cycle arrest that we obtained were similar to the published analyses of cell cycle delays. Based on linear regression analysis, we found no relationship between the degree of $G_2$ and $G_1$ arrests ($r=-0.146$, $P=0.730$), and conclude that the two blocks are independent events that can be assessed by PI staining. We found two different trends in the cell lines studied. Some cells were arrested in $G_1$, and we presume that they probably had wild-type p53 (Kastan *et al.*, 1991). In fact, they have low endogenous p53 levels, which may be an indirect indication of p53 functionality. Cell lines MCF-7 BUS, MCF-7 GS and D283MED may also belong to this group. In contrast the rest of the cell lines (MCF-7 BB, T47D, MDA-MB-231 EVSA-T and RT-112) were arrested in $G_2$ but not in $G_1$, and probably correspond to cells with non-functional

**Figure 2** Time course of $G_2$/M ratio for irradiated (6 Gy) *vs* unirradiated cells, assessed by flow cytometry. Points represents means of at least three experiments; a minimum of 10 000 events were counted.

p53. This may correlate with the higher p53 levels observed in these lines (Kastan *et al.*, 1991). Cell line MCF-7 has wild-type p53 (Takahashi and Suzuki, 1993), whereas T47D and MDA-MB-231 have mutant p53 (Bartek *et al.*, 1990).

We found a close relationship between intrinsic cellular radiosensitivity and the degree of $G_1$ arrest observed ($r=-0.869$, $P=0.0051$). In contrast, our data do not support the relationship between $G_2$ arrest and radiosensitivity (Figure 3).

### *P53 protein ELISA whole cell assay*

The relationship between optical density (OD) measured at 492 nm and cell number was linear in all experiments. The *P*-values of this relationship were always highly significant ($P<0.0001$). When the p53 values in OD units were plotted on the *y*-axes *vs* cell number, the straight lines corresponding to each cell line differed widely in their slope (Figure 4, Table I). Each experiment was done at least three times, and the results obtained suggest that the assay was highly reproducible. Background levels of OPD staining were typically about 0.065 OD units. Corresponding background values were subtracted in each experiment.

To validate the ELISA whole cell assay we used HL-60 myeloid leukaemia cells, which lack endogenous p53 genes (Kuerbitz *et al.*, 1992). In this experiment the values of p53 OD were independent of cell number, and did not show any differences between the values for signal or noise (slope = 0.00, Table I). Overall we found a close relationship between the constitutive levels of p53 and the SF2 values ($r=0.874$, $P=0.0046$, Figure 5a) in the panel of cell lines used. Cells with the highest slopes were the most radioresistant, whereas lower slope values corresponded to radiosensitive cells. The high levels of p53 in radioresistant lines may be an indirect indication that these lines contain non-functional p53 protein.

### *Time course of p53 induction*

We determined intracellular p53 levels at different times after cellular irradiation. There were two extreme patterns of response: (1) in lines MCF-7 BUS, MCF-7 GS and D283MED, there is an initial increase in p53 intracellular levels, which reached maximum values 4 h after irradiation; (2) in lines MCF-7 BB, T47D, EVSA-T, MDA-MB-231 and RT-112, p53 showed little or no response of p53 to DNA damage induced by radiation. Figure 6a shows an example from each group.

These time course patterns were confirmed by p53 Western blotting assays (Figure 7). We chose 4 h after cell irradiation as a reference point to study the p53 response to different doses of radiation. These experiments revealed differences between the cell lines that seemed to correlate with one or other of the patterns described above (Figure 6b). The mean values of p53 fold induction 4 h after treatment are shown in Table I. Interestingly, there was a close relationship between the level of p53 fold induction and both intrinsic cellular radiosensitivity (SF2), ($r=0.882$, $P=0.0038$, Figure 5b) and the degree of $G_1$ arrest ($r=0.889$, $P=0.0032$, Figure 5c).

### *Apoptotic response to γ-radiation*

Chromatin cleavage appears to be the most characteristic biochemical feature of the apoptotic process. The appearance of the ladder of nucleosomal DNA fragments in agarose gels is thus the hallmark of apoptosis. We assessed apoptosis 24 and 48 h after treatment with 6 Gy, and assigned one of three possible scores to each cell line (Figure 8, Table I). MCF-7 BUS, MCF-7 GS and D283MED were classified as positive (class +: clear appearance of oligonucleosomal

**Figure 3** Radiation-induced cell cycle arrest and radiosensitivity. (**a**) Surviving fraction at 2 Gy and maximal degree of $G_1$ arrest (24–30 h), $r=-0.869$, $P=0.0051$. (**b**) Surviving fraction at 2 Gy and maximal degree of $G_2$ arrest (12–18 h), $r=0.284$, $P=0.495$. The percentage of cells was referred to the values in the controls and expressed as the relative proportion of cells in $G_1$ and $G_2$. Points are means of at least three experiments ± s.e.m.

**Figure 4** Immunoenzyme assay to quantify p53 levels in MDA-MB-231 (■) and MCF-7 BUS (●) cell lines. Cells were plated at densities of 25000–150000 cells per well and optical densities (OD) were measured in a plate reader at 492 nm. Points are means of at least three experiments ± s.e.m.

**Figure 5** (**a**) Relationship between constitutive levels of p53 and surviving fraction at 2 Gy, $r = 0.874$, $P = 0.0046$. (**b**) Relationship between fold induction of p53 4 h after cell treatment with $\gamma$-rays and surviving fraction at 2 Gy, $r = -0.882$, $P = 0.0038$. (**c**) Relationship between fold induction of p53 4 h after cell treatment with $\gamma$-rays and degree of $G_1$ arrest, $r = 0.889$, $P = 0.0032$. Points are means of at least three experiments ± s.e.m.

fragments); lines RT-112 and EVSA-T showed a trace amount of DNA ladder formation (class ±); and lines MDA-MB-231, MCF-7 BB and T47D were negative (class –, smear pattern).

**Figure 6** (**a**) Time-course of p53 response to irradiation. (**b**) p53 response 4 h after cell treatment with different doses of radiation. Each point represents the mean of two independent experiments performed by quadruplicate ± s.e.m. MCF-7 BUS (■) and MDA-MB-231 (□).

**Figure 7** (**a**) Levels of p53 protein measured by Western blotting at various times after ionising radiation. (**b**) Relative intensity of different bands quantified by image analysis in D283MED (●) and RT-112 (○).

**Figure 8** DNA analysis by 1% agarose gel electrophoresis of genomic DNA extracted from D283MED (+, clear appearance of oligonucleosomal fragments), EVSA-T (±, trace amount of DNA ladder formation) and MCF-7 BB (−, smear pattern) 48 h after irradiation.

### Discussion

It has been realised for some time that human tumour cell lines can differ widely in their survival characteristics after treatment with ionising radiation. The data presented here are representative of the range of radiosensitivities (0.18–0.82) commonly seen in human tumour cell lines. Studies of DNA removed from cells immediately after irradiation reveal extensive damage, and it is generally accepted that ionising radiation kills eukaryotic cells by damaging the structure and function of genomic DNA. Recent evidence suggests that DNA damage causes transient alterations in cell cycle progression via both $G_1$ and $G_2$ arrests (Kastan *et al*., 1991). Differences in cell cycle arrests have been shown to be associated with quantifiable differences in cellular radiosensitivity (Kastan *et al*., 1991, 1992; Canman *et al*., 1994; Kuerbitz *et al*., 1992; McIlwrath *et al*., 1994; Fan *et al*., 1994; O'Connor *et al*., 1993; Nagasawa *et al*., 1994). Until recently the association of prolonged cell cycle delays with radioresistance was interpreted as a means by which the cell is given increased time to repair DNA damage (Kastan *et al*., 1991). It is now recognised that p53 plays a key role in the $G_1$/S transition through its transactivation of WAF1/Cip1, which inhibits $G_1$ cyclin-dependent kinases (Harper *et al*., 1993; El-Deiry *et al*., 1993).

Studies of p53 have suggested that the above interpretation of the importance of the post irradiation checkpoints may be inadequate. It was recently suggested that p53 protein is involved in DNA damage recognition and apoptosis initiation. Thus p53 gene status and cellular radiosensitivity might be connected. Mutant p53 has been shown to decrease the radiation-induced $G_1$ arrest but to increase radioresistance (McIlwrath *et al*., 1994; Fan *et al*., 1994; O'Connor *et al*., 1993). This has been explained in some systems by the requirement for functional p53 to be present for apoptosis to occur (Lowe *et al*., 1993; Merritt *et al*., 1994), but it is not clear whether this is always the route by which p53 alters radiosensitivity. Xia (1995) has recently reported a correlation between altered p53 status, high p53 constitutive levels, reduced increase in p53 levels after irradiation and radioresistance in two lymphocyte lines, but there was no difference in the overall degree of apoptosis.

The possible relationship between p53 mutation and radiosensitivity has obvious implications for radiotherapy (Lowe *et al*., 1994; Levine *et al*., 1994), because of the high incidence of p53 mutations in human cancers. This is the issue that the present study was designed to address. A study by McIlwrath *et al*. (1994) suggested that there are two groups of tumour cell lines, based on p53 function assessed by p53 induction by radiation and suppression of DNA synthesis. The data presented here confirm this finding in a different set of human tumour cell lines, and document a close overall correlation between radiosensitivity, constitutive p53 levels, the degree of p53 induction and modifications in the cell cycle $G_1$ checkpoint. Although a correlation is not proof of a cause, the relationship seen here is close enough to strongly imply that p53 function is an important determinant of radiosensitivity.

To date, we have investigated apoptosis (by DNA fragmentation assay) in all cell lines tested here, and have found a close relationship between the appearance of oligonucleosomal fragments and $G_0$/$G_1$ cell cycle arrest (Table I). Moreover, a smear pattern or a trace amount of DNA fragmentation are common findings in cells containing non-functional p53. It has been proposed that p53-dependent apoptosis is a cell type-specific phenomenon, and that the $G_2$ checkpoint may also be important in determining radiosensitivity (Slichenmyer *et al*., 1993). In this connection, although our results support the idea of the greater importance of the $G_1$/S boundary in relation to radiosensitivity, we cannot exclude a role for the $G_2$/M checkpoint as a determinant of the response in cells that do not show $G_0$/$G_1$ arrest. In fact, although loss of wild type p53 may abrogate $G_1$ arrest, radiation-induced apoptosis can still occur in human tumour cell lines through a mechanism independent of p53 (Bracey *et al*., 1995). We found a weak ladder pattern in RT-112 and EVSA-T cells, both of which show no $G_1$ arrest. To elucidate the importance of apoptosis for intrinsic cellular radiosensitivity apoptosis must be studied quantitatively. We have investigated apoptosis in cell line D283MED (Ung *et al*., in preparation), and have found that despite its apparently normal p53 response, apoptosis occurs in a minority of cells even after a dose that reduces survival to 0.001. Thus, although apoptosis may be a factor in some of the cell lines described here, it appears unlikely to be the only explanation for the high sensitivity of cells with an apparently intact p53 system.

In conclusion, the use of different tests based on (1) the presence of functional $G_1$ arrest after cell treatment (Figure 3a); (2) the quantitative measurement of constitutive levels of p53 protein in the tumour cells (Figure 5a); and (3) the increase in intracellular p53 levels after DNA radiation-induced damage (Figure 5b), could offer a solution to the problem of the assessment of intrinsic radiosensitivity as a predictor of patient response to radiotherapy. However, further evidence in support of this hypothesis may well come from studies of the roles of p53, cell cycle control mechanisms and the relative importance of apoptosis and mitotic cell death after irradiation, which are now being pursued at our laboratory.

**Acknowledgements**
This work was supported by the Comisión Interministerial de Ciencia y Tecnología (CICYT) through the project SAF 95-778. A grant from the the Fundación Científica de la Asociación Española Contra el Cáncer greatly aided this work. E Siles is supported by grant PN93 26004172 and MT Valenzuela by grant PN 92 8837784 from the Spanish Ministry of Eduction and Science. A Gordon and TJ McMillan are supported by the Cancer Research Campaign, the Medical Research Council and the Association for International Cancer Research. We thank Professor GG Steel and Dr J Peacock for comments on the manuscript and Dr MA Lucena for help with flow cytometry. Our thanks also to Francisca Gutierrez for secretarial help and Karen Shaskok for improving the use of English in the manuscript.

### References

BAKER SJ, MARKOWITZ S, FEARON ER, WILLSON JKV AND VOGELSTEIN B. (1990). Suppression of human colorectal carcinoma cell growth by wild-type p53. *Science*, **249**, 912–915.

BARTEK J, IGGO R, GANNON J AND LANE DP. (1990). Genetic and immunochemical analysis of mutant p53 in human breast cancer cell lines. *Oncogene*, **5**, 893–899.

BRACEY TS, MILLER JC, PREECE A AND PARASKEVA C. (1995). $\gamma$-Radiation-induced apoptosis in human colorectal adenoma and carcinoma cell lines can occur in the absence of wild type p53. *Oncogene*, **10**, 2391–2396.

CAILLEAU R, YOUNG R, OLIVE M AND REEVES Jr WJ. (1974). Breast tumour cell lines from pleural effusions. *J. Natl Cancer Inst.*, **53**, 661–666.

CANMAN CE, WOLFF AC, CHEN CY, FORNACE Jr AJ AND KASTAN MB. (1994). The p53-dependent $G_1$ cell cycle checkpoint pathway and ataxia-telangiectasia. *Cancer Res.*, **54**, 5054–5058.

COLLINS SJ, GALLO RC AND GALLAGHER RE. (1977). Continuous growth and differentiation of human myeloid cells in suspension culture. *Nature*, **270**, 347–349.

EL-DEIRY WS, TOKINO T, VELCULESCU VE, LEVY DB, PARSONS R, TRENT JM, LIN D, MERCER WE, KINZLER KW AND VOGELSTEIN B. (1993). WAF1, a potential mediator of p53 tumour suppression. *Cell*, **75**, 817–825.

FAN S, EL-DEIRY WS, BAE I, FREEMAN J, JONDLE D, BHATIA K, FORNACE Jr AJ, MAGRATH I, KOHN KW AND O'CONNOR P. (1994). p53 gene mutations are associated with decreased sensitivity of human lymphoma cells to DNA damaging agents. *Cancer Res.*, **54**, 5824–5830.

FRIEDMAN HS, BURGER PC, BIGNER SC, TROJANOWSKI J, HALPERIN EC AND BIGNER DD. (1985). Establishment and characterisation of the human medulloblastoma cell line and transplantable xenograft D283 MED. *J. Neuropathol. Exp. Neurol.*, **44**, 592–605.

HALL PA, RAY A, LEMOINE NR, MIDGLEY CA, KRAUSZT T AND LANE DP. (1991). p53 immunostaining as a marker of malignant disease in diagnostic cytopathology. *Lancet*, **338**, 513.

HARPER JW, ADAMI GR, WEI N, KEYOMARS K AND ELLEDGE SJ. (1993). The p21 Cdk-interacting protein Cip1 is a potent inhibitor of $G_1$ cyclin-dependent kinases. *Cell*, **75**, 805–816.

HOLLSTEIN M, SIDRANSKY D, VOGELSTEIN B AND HARRIS CC. (1991). p53 mutations in human cancers. *Science*, **253**, 49–53.

KASTAN MB, ONYEKWERE O, SIDRANSKY D, VOGELSTEIN B AND CRAIG RW. (1991). Participation of p53 protein in the cellular response to DNA damage. *Cancer Res.*, **51**, 6304–6311.

KASTAN MB, ZHAN Q, EL-DEIRY WS, CARRIER F, JACKS T, WALSH WV, PLUNKETT BS, VOGELSTEIN B AND FORNACE Jr AJ. (1992). A mammalian cell cycle checkpoint pathway utilizing p53 and Gadd45 is defective in ataxia-telangiectasia. *Cell*, **71**, 587–597.

KEYDAR I, CHEN L, KARBY S, WEISS FR, DELAREA J, RADU M, CHAITIK S AND BRENNER HJ. (1979). Establishment and characterization of a cell line of human breast carcinoma origin. *Eur. J. Cancer*, **15**, 659–670.

KUERBITZ SJ, PLUNKETT BS, WALSH WV AND KASTAN MB. (1992). Wild-type p53 is a cell cycle checkpoint determinant following irradiation. *Proc. Natl Acad. Sci. USA*, **89**, 7491–7495.

LEVINE AJ, MOMAND J AND FINLAY CA. (1991). The p53 tumour suppressor gene. *Nature*, **351**, 453–456.

LEVINE AJ, PERRY ME, CHANG A, SILVER A, DITTMER D, WU M AND WELSH D. (1994). The 1993 Walter Hubert Lecture: The role of the p53 tumour-suppressor gene in tumorigenesis. *Br. J. Cancer*, **69**, 409–416.

LIPPMAN M, BOLAN G AND HUFF K. (1976). The effects of estrogen and antiestrogens on hormone-responsive human breast cancer in long-term tissue culture. *Cancer Res.*, **36**, 4595–4601.

LOWE SW, SCHMITT EM, SCHMITT SW, OSBORNE BA AND JACKS T. (1993). p53 is required for radiation-induced apoptosis in mouse thymocytes. *Nature*, **362**, 847–849.

LOWE SW, BODIS S, MCCLATCHEY A, REMINGTON L, RULEY HE, FISHER DE, HOUSEMAN DE AND JACKS T. (1994). p53 status and the efficacy of cancer therapy in vivo. *Science*, **266**, 807–810.

MCILWRATH AJ, VASEY PA, ROSS GM AND BROWN R. (1994). Cell cycle arrests and radiosensitivity of human tumour cell lines: dependence on wild-type p53 for radiosensitivity. *Cancer Res.*, **54**, 3718–3722.

MASTERS JRW, HEPBURN PJ, WALKER L, HIGHMAN WJ, TREJDOSIEWICZ LK, POVEY S, HILL BT, RIDDLE PR AND FRANKS LM. (1986). Tissue culture models of transitional cell carcinoma: characterization of 22 human urothelial cell lines. *Cancer Res.*, **4**, 3630–3636.

MERRITT AJ, POTTEN CS, KEMP J, HICKMAN JA, BALMAIN A, LANE DP AND HALL PA. (1994). The role of p53 in spontaneous and radiation-induced apoptosis in the gastrointestinal tract of normal and p53-deficient mice. *Cancer Res.*, **54**, 614–617.

NAGASAWA H, KENG P, HARLEY R, DAHLBERG W AND LITTLE JB. (1994). Relationship between gamma ray-induced $G_2$/M delay and cellular radiosensitivity. *Int. J. Radiat. Biol.*, **66**, 373–379.

NÚÑEZ MI, VILLALOBOS M, OLEA N, VALENZUELA MT, PEDRAZA V, MCMILLAN TJ AND RUIZ DE ALMODÓVAR JM. (1995). Radiation-induced DNA double-strand break rejoining in human tumour cells. *Br. J. Cancer*, **71**, 311–316.

O'CONNOR PM, JACKMAN J, JONDLE D, BHATIA K, MAGRATH I AND KOHN K. (1993). Role of p53 tumor suppressor gene in cell cycle arrest and radiosensitivity of Burkitt's lymphoma cell lines. *Cancer Res.*, **53**, 4776–4780.

POWELL SN AND MCMILLAN TJ. (1994). The repair fidelity of restriction enzyme-induced double strand breaks in plasmid DNA correlates with radioresistance in human tumor cell lines. *Int. J. Rad. Oncol. Biol. Phys.*, **29**, 1035–1040.

ROBINSON JP. (1993). Measurements of DNA. In *Handbook of Flow Cytometry Methods*, Robinson JP (ed.), Darzynkiewicz Z, Dean P, Dressler L, Tanke H and Wheeless L. (assoc eds) pp. 90–126. Wiley-Liss: New York.

RUIZ DE ALMODÓVAR JM, NÚÑEZ MI, MCMILLAN TJ, OLEA N, MORT C, VILLALOBOS M, PEDRAZA V AND STEEL GG. (1994). Initial DNA damage is a determinant of intrinsic cellular radiosensitivity. *Br. J. Cancer*, **69**, 457–462.

SCHLICHTHOLZ B, LEGROS Y, GILLET D, GAILLARD C, MARTY M, LANE D, CALVO F AND SOUSSI T. (1992). The immune response to p53 in breast cancer patients is directed against immunodominant epitopes unrelated to the mutational hot spot. *Cancer Res.*, **52**, 6380–6384.

SLICHENMYER WJ, NELSON WG, SLEBOS RJ AND KASTAN MB. (1993). Loss of a p53-associated $G_1$ checkpoint does not decrease cell survival following DNA damage. *Cancer Res.*, **53**, 4164–4168.

SOTO AM, MURAI JT, SITERI PK AND SONNENSCHEIN C. (1986). Control of cell proliferation: evidence for negative control on estrogen-sensitive T47D human breast cancer cells. *Cancer Res.* **46**, 2271–2275.

SOULE D, VAZQUEZ J, LONG A, ALBERT S AND BRENNAN M. (1973). Human cell line from a pleural effusion derived from a breast carcinoma. *J. Natl Cancer Inst.*, **51**, 1409–1413.

STEWART N, HICKS GG, PARASKEVAS F AND MOWAT M. (1995). Evidence for a second cell cycle block at G2/M by p53. *Oncogene*, **10**, 109–115.

STRASSER A, HARRIS AW, JACKS T AND CORY S. (1994). DNA damage can induce apoptosis in proliferating lymphoid cells via p53-independent mechanisms inhibitable by Bcl-2. *Cell*, **72**, 329–339.

TAKAHASHI K AND SUZUKI K. (1993). Association of insulin-like growth-factor-I-induced DNA synthesis with phosphorylation and nuclear exclusion of p53 in human breast cancer MCF-7 cells. *Int. J. Cancer*, **55**, 453–458.

WHITAKER SJ, UNG YC AND MCMILLAN TJ. (1995). DNA double strand break induction and rejoining as determinants of human tumour cell radiosensitivity. A pulsed-field gel electrophoresis study. *Int. J. Radiat. Biol.*, **67**, 1, 7–18.

WURM R, BURNET NG, DUGGAL N, YARNOLD JR AND PEACOCK JH. (1995). Cellular radiosensitivity and DNA damage in primary human fibroblasts. *Int. J. Radiat. Oncol. Biol. Phys.*, **30**, 3, 625–633.

XIA F, WANG X, WANG YH, TSANG NM, YANDELL DW, KELSEY KT AND LIBER HL. (1995). Altered p53 status correlates with differences in sensitivity to radiation-induced mutation and apoptosis in two closely related human lymphoblast lines. *Cancer Res.*, **55**, 12–15.


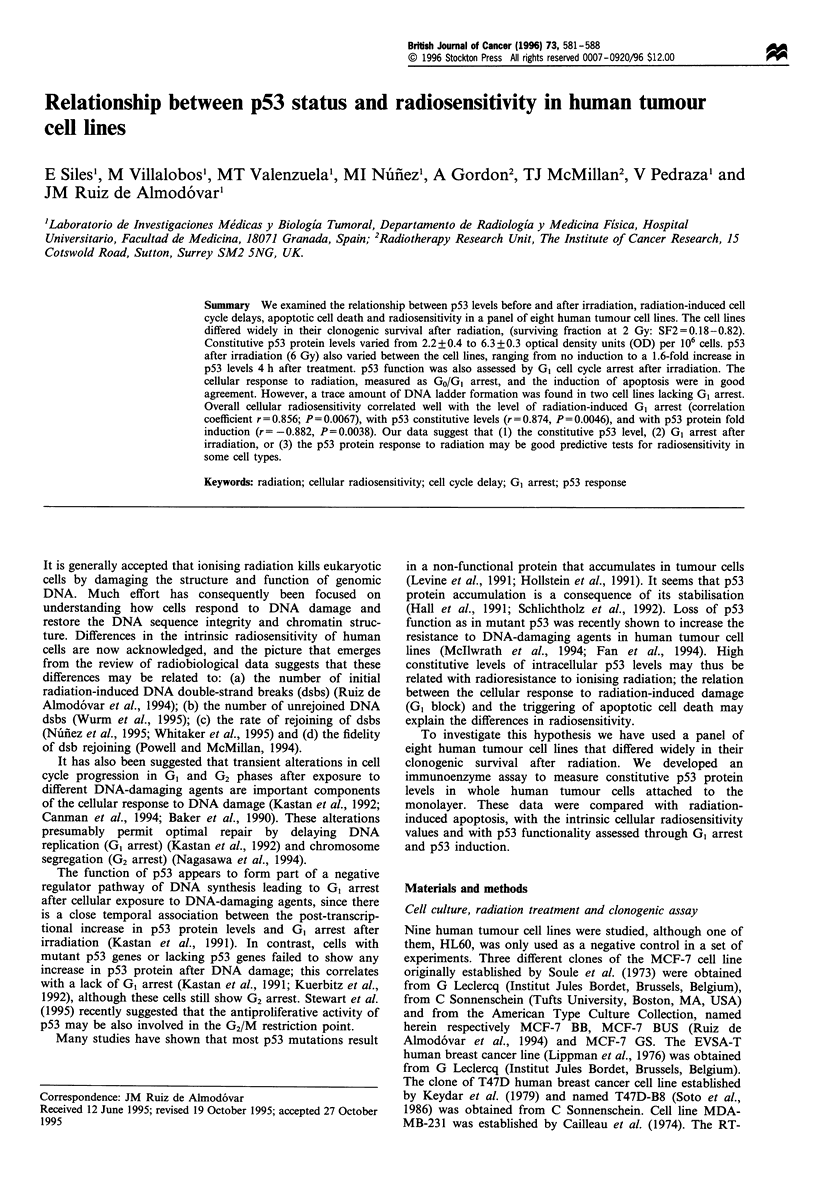

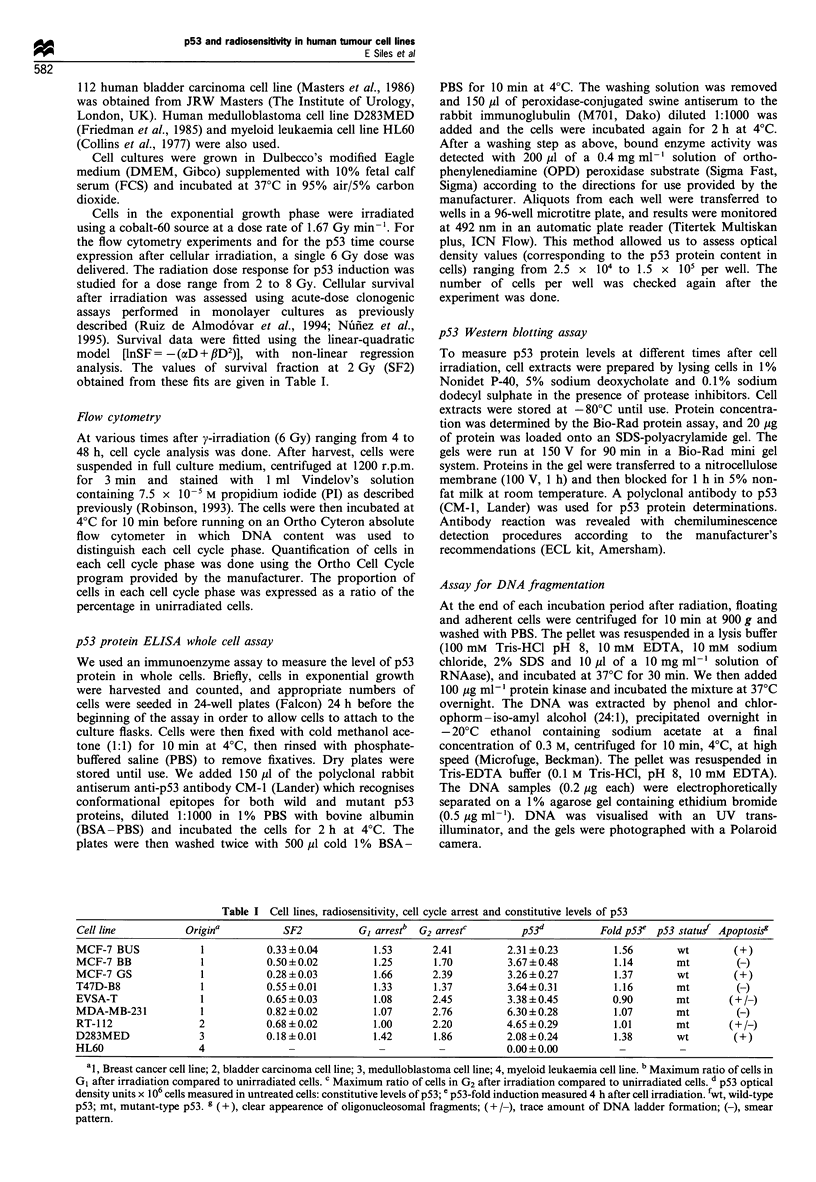

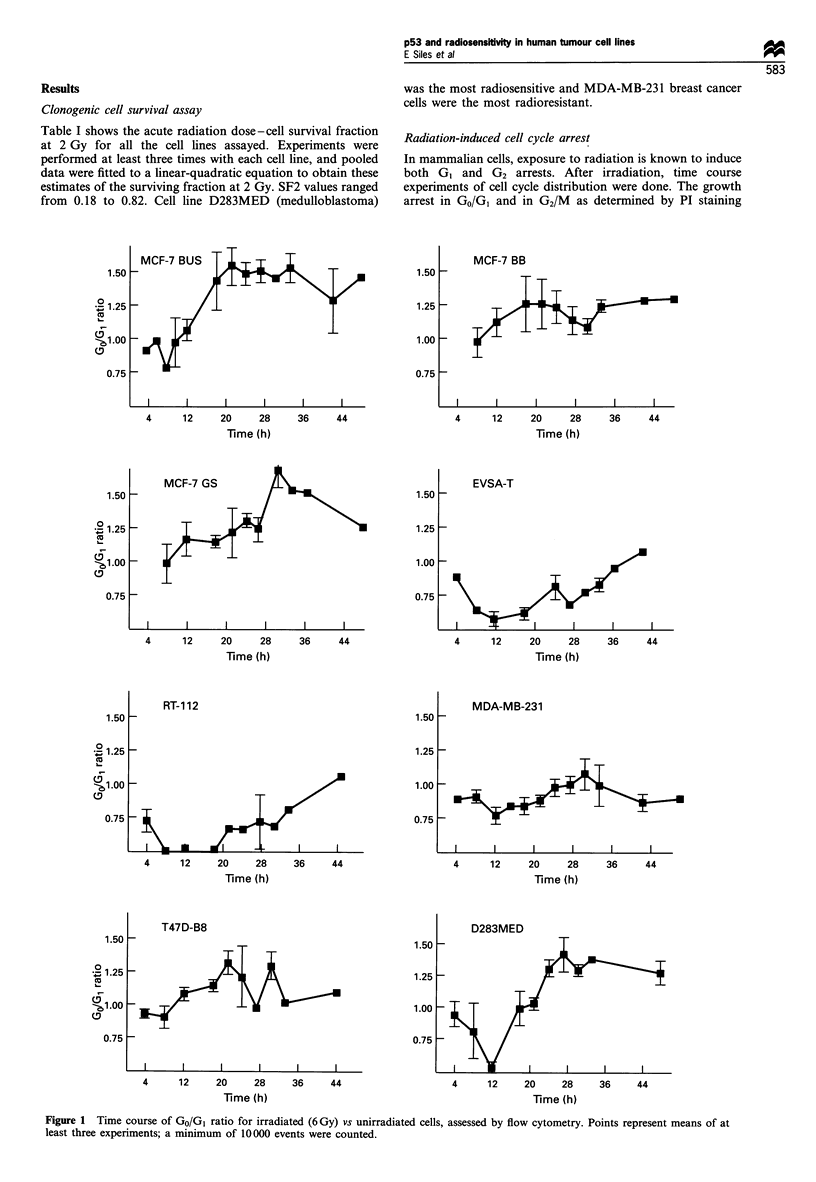

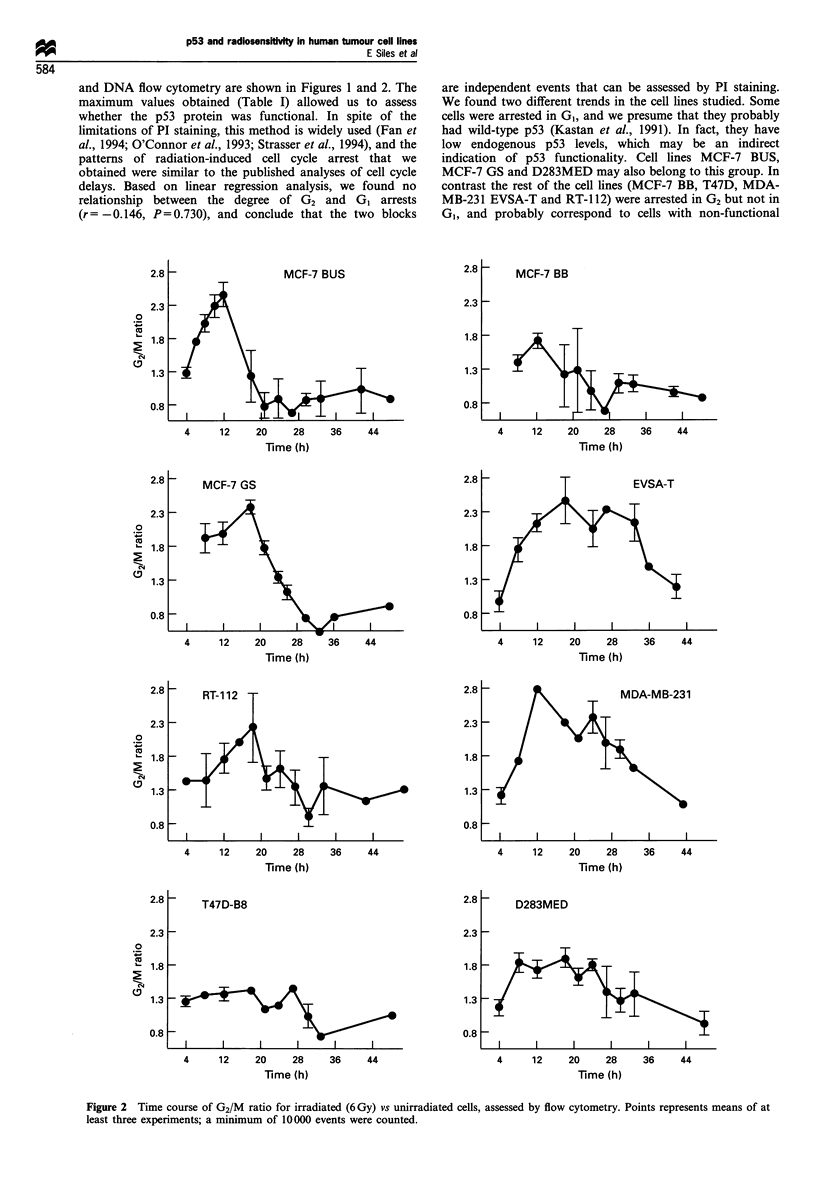

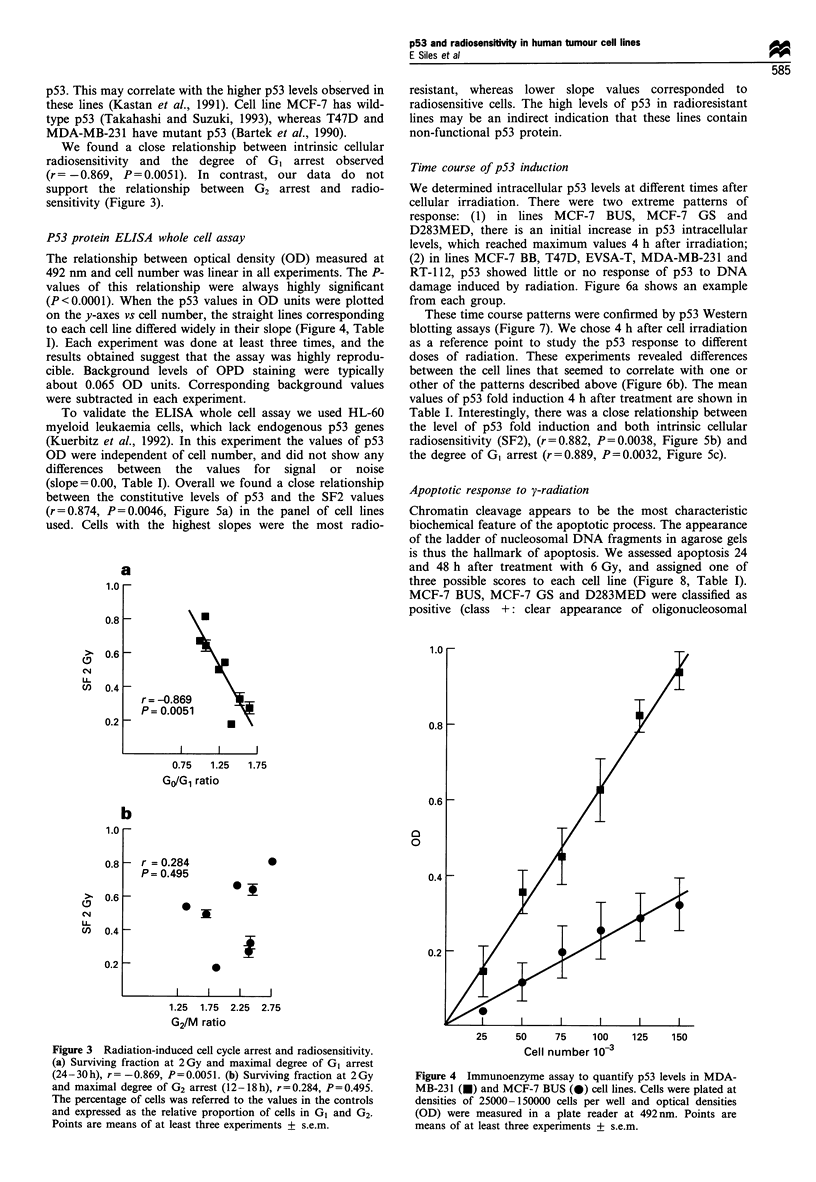

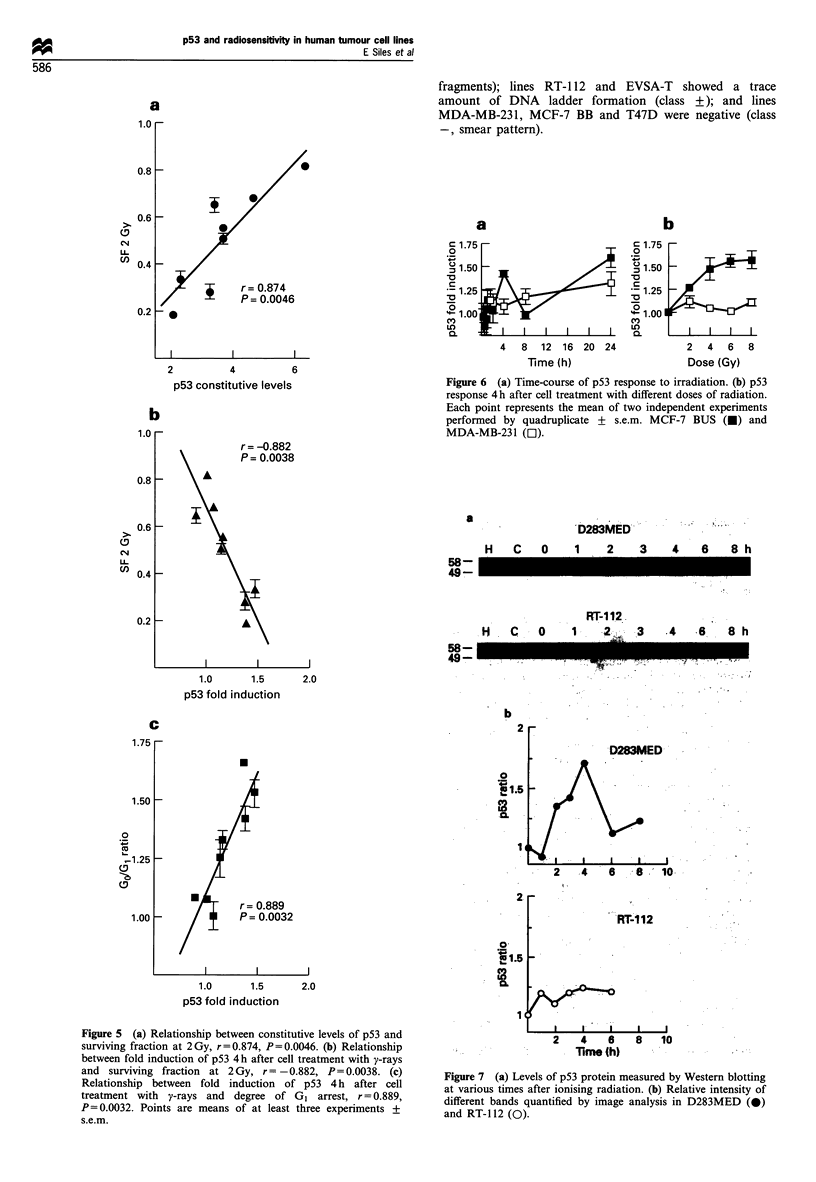

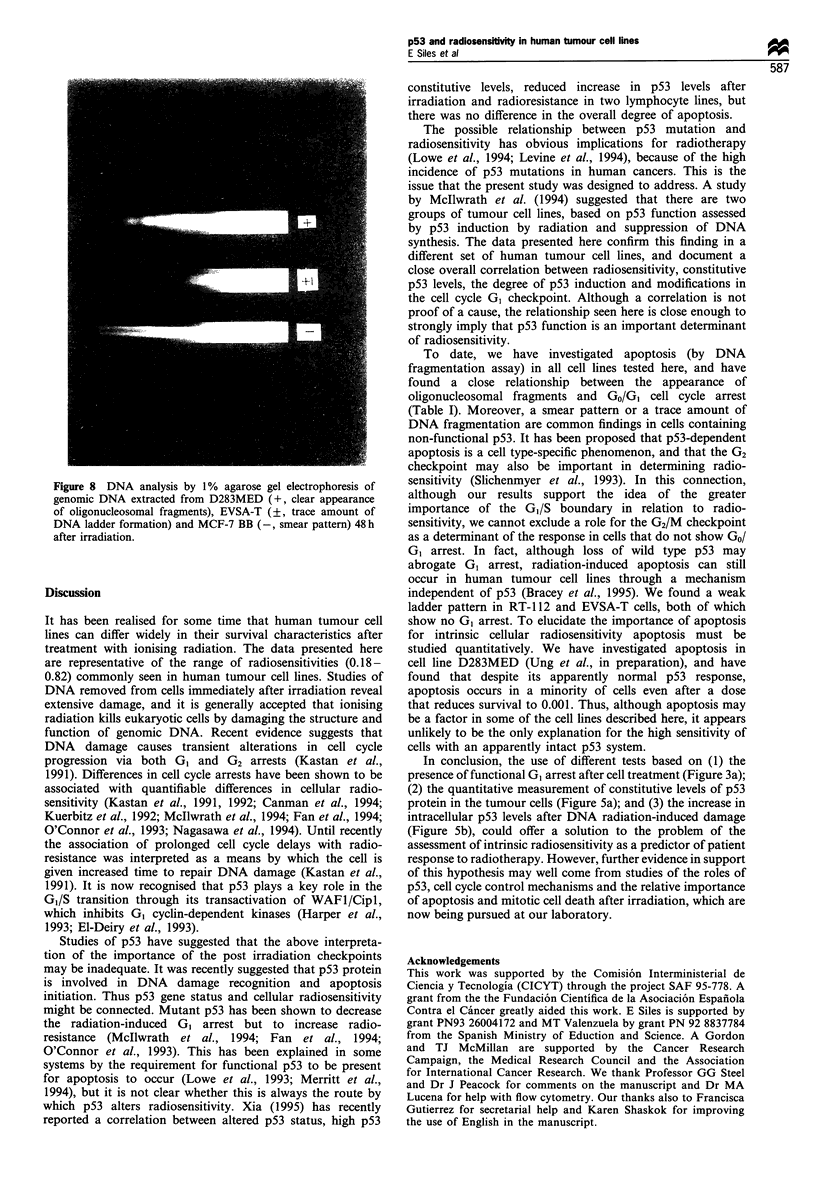

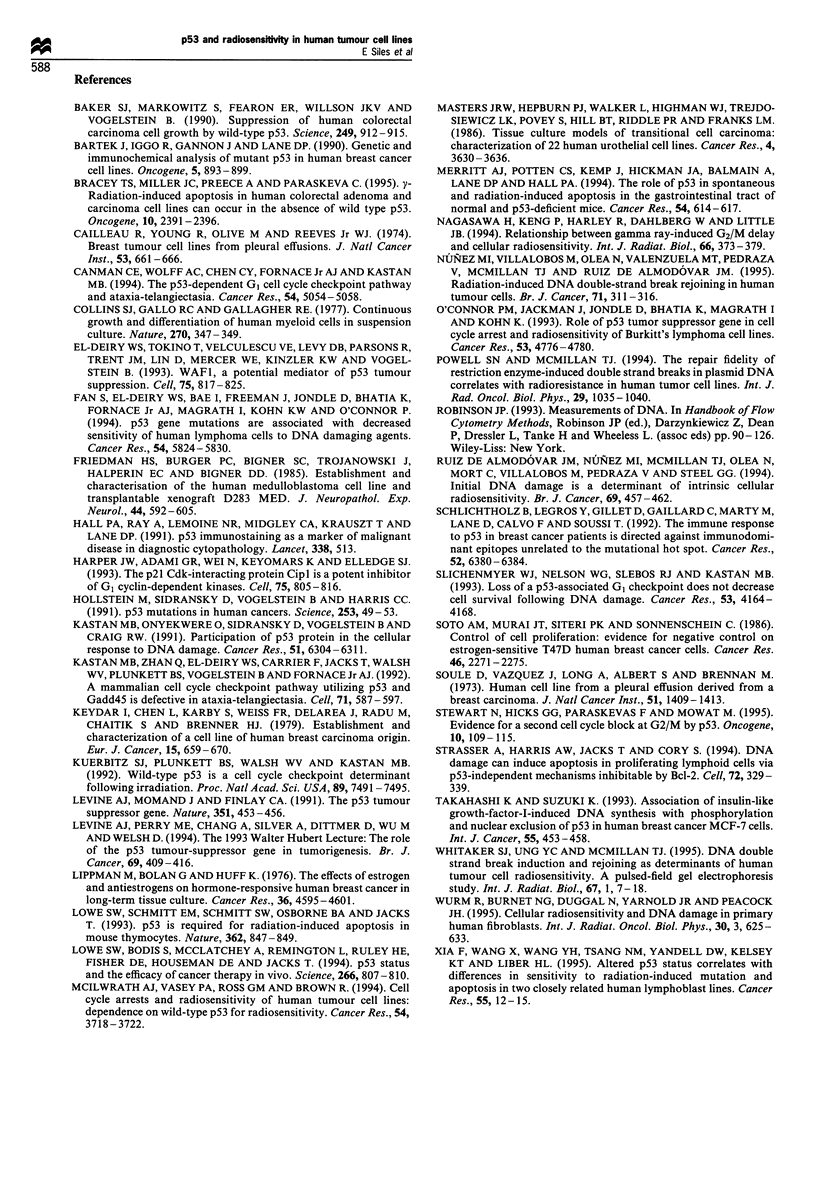


## References

[OCR_01120] Baker S. J., Markowitz S., Fearon E. R., Willson J. K., Vogelstein B. (1990). Suppression of human colorectal carcinoma cell growth by wild-type p53.. Science.

[OCR_01125] Bartek J., Iggo R., Gannon J., Lane D. P. (1990). Genetic and immunochemical analysis of mutant p53 in human breast cancer cell lines.. Oncogene.

[OCR_01130] Bracey T. S., Miller J. C., Preece A., Paraskeva C. (1995). Gamma-radiation-induced apoptosis in human colorectal adenoma and carcinoma cell lines can occur in the absence of wild type p53.. Oncogene.

[OCR_01138] Cailleau R., Young R., Olivé M., Reeves W. J. (1974). Breast tumor cell lines from pleural effusions.. J Natl Cancer Inst.

[OCR_01141] Canman C. E., Wolff A. C., Chen C. Y., Fornace A. J., Kastan M. B. (1994). The p53-dependent G1 cell cycle checkpoint pathway and ataxia-telangiectasia.. Cancer Res.

[OCR_01148] Collins S. J., Gallo R. C., Gallagher R. E. (1977). Continuous growth and differentiation of human myeloid leukaemic cells in suspension culture.. Nature.

[OCR_01157] Fan S., el-Deiry W. S., Bae I., Freeman J., Jondle D., Bhatia K., Fornace A. J., Magrath I., Kohn K. W., O'Connor P. M. (1994). p53 gene mutations are associated with decreased sensitivity of human lymphoma cells to DNA damaging agents.. Cancer Res.

[OCR_01167] Friedman H. S., Burger P. C., Bigner S. H., Trojanowski J. Q., Wikstrand C. J., Halperin E. C., Bigner D. D. (1985). Establishment and characterization of the human medulloblastoma cell line and transplantable xenograft D283 Med.. J Neuropathol Exp Neurol.

[OCR_01171] Hall P. A., Ray A., Lemoine N. R., Midgley C. A., Krausz T., Lane D. P. (1991). p53 immunostaining as a marker of malignant disease in diagnostic cytopathology.. Lancet.

[OCR_01176] Harper J. W., Adami G. R., Wei N., Keyomarsi K., Elledge S. J. (1993). The p21 Cdk-interacting protein Cip1 is a potent inhibitor of G1 cyclin-dependent kinases.. Cell.

[OCR_01183] Hollstein M., Sidransky D., Vogelstein B., Harris C. C. (1991). p53 mutations in human cancers.. Science.

[OCR_01188] Kastan M. B., Onyekwere O., Sidransky D., Vogelstein B., Craig R. W. (1991). Participation of p53 protein in the cellular response to DNA damage.. Cancer Res.

[OCR_01192] Kastan M. B., Zhan Q., el-Deiry W. S., Carrier F., Jacks T., Walsh W. V., Plunkett B. S., Vogelstein B., Fornace A. J. (1992). A mammalian cell cycle checkpoint pathway utilizing p53 and GADD45 is defective in ataxia-telangiectasia.. Cell.

[OCR_01196] Keydar I., Chen L., Karby S., Weiss F. R., Delarea J., Radu M., Chaitcik S., Brenner H. J. (1979). Establishment and characterization of a cell line of human breast carcinoma origin.. Eur J Cancer.

[OCR_01202] Kuerbitz S. J., Plunkett B. S., Walsh W. V., Kastan M. B. (1992). Wild-type p53 is a cell cycle checkpoint determinant following irradiation.. Proc Natl Acad Sci U S A.

[OCR_01208] Levine A. J., Momand J., Finlay C. A. (1991). The p53 tumour suppressor gene.. Nature.

[OCR_01210] Levine A. J., Perry M. E., Chang A., Silver A., Dittmer D., Wu M., Welsh D. (1994). The 1993 Walter Hubert Lecture: the role of the p53 tumour-suppressor gene in tumorigenesis.. Br J Cancer.

[OCR_01216] Lippman M., Bolan G., Huff K. (1976). The effects of estrogens and antiestrogens on hormone-responsive human breast cancer in long-term tissue culture.. Cancer Res.

[OCR_01229] Lowe S. W., Bodis S., McClatchey A., Remington L., Ruley H. E., Fisher D. E., Housman D. E., Jacks T. (1994). p53 status and the efficacy of cancer therapy in vivo.. Science.

[OCR_01223] Lowe S. W., Schmitt E. M., Smith S. W., Osborne B. A., Jacks T. (1993). p53 is required for radiation-induced apoptosis in mouse thymocytes.. Nature.

[OCR_01238] Masters J. R., Hepburn P. J., Walker L., Highman W. J., Trejdosiewicz L. K., Povey S., Parkar M., Hill B. T., Riddle P. R., Franks L. M. (1986). Tissue culture model of transitional cell carcinoma: characterization of twenty-two human urothelial cell lines.. Cancer Res.

[OCR_01232] McIlwrath A. J., Vasey P. A., Ross G. M., Brown R. (1994). Cell cycle arrests and radiosensitivity of human tumor cell lines: dependence on wild-type p53 for radiosensitivity.. Cancer Res.

[OCR_01246] Merritt A. J., Potten C. S., Kemp C. J., Hickman J. A., Balmain A., Lane D. P., Hall P. A. (1994). The role of p53 in spontaneous and radiation-induced apoptosis in the gastrointestinal tract of normal and p53-deficient mice.. Cancer Res.

[OCR_01251] Nagasawa H., Keng P., Harley R., Dahlberg W., Little J. B. (1994). Relationship between gamma-ray-induced G2/M delay and cellular radiosensitivity.. Int J Radiat Biol.

[OCR_01256] Núez M. I., Villalobos M., Olea N., Valenzuela M. T., Pedraza V., McMillan T. J., Ruiz de Almodóvar J. M. (1995). Radiation-induced DNA double-strand break rejoining in human tumour cells.. Br J Cancer.

[OCR_01262] O'Connor P. M., Jackman J., Jondle D., Bhatia K., Magrath I., Kohn K. W. (1993). Role of the p53 tumor suppressor gene in cell cycle arrest and radiosensitivity of Burkitt's lymphoma cell lines.. Cancer Res.

[OCR_01268] Powell S. N., McMillan T. J. (1994). The repair fidelity of restriction enzyme-induced double strand breaks in plasmid DNA correlates with radioresistance in human tumor cell lines.. Int J Radiat Oncol Biol Phys.

[OCR_01281] Ruiz de Almodóvar J. M., Núez M. I., McMillan T. J., Olea N., Mort C., Villalobos M., Pedraza V., Steel G. G. (1994). Initial radiation-induced DNA damage in human tumour cell lines: a correlation with intrinsic cellular radiosensitivity.. Br J Cancer.

[OCR_01287] Schlichtholz B., Legros Y., Gillet D., Gaillard C., Marty M., Lane D., Calvo F., Soussi T. (1992). The immune response to p53 in breast cancer patients is directed against immunodominant epitopes unrelated to the mutational hot spot.. Cancer Res.

[OCR_01293] Slichenmyer W. J., Nelson W. G., Slebos R. J., Kastan M. B. (1993). Loss of a p53-associated G1 checkpoint does not decrease cell survival following DNA damage.. Cancer Res.

[OCR_01297] Soto A. M., Murai J. T., Siiteri P. K., Sonnenschein C. (1986). Control of cell proliferation: evidence for negative control on estrogen-sensitive T47D human breast cancer cells.. Cancer Res.

[OCR_01303] Soule H. D., Vazguez J., Long A., Albert S., Brennan M. (1973). A human cell line from a pleural effusion derived from a breast carcinoma.. J Natl Cancer Inst.

[OCR_01310] Stewart N., Hicks G. G., Paraskevas F., Mowat M. (1995). Evidence for a second cell cycle block at G2/M by p53.. Oncogene.

[OCR_01315] Strasser A., Harris A. W., Jacks T., Cory S. (1994). DNA damage can induce apoptosis in proliferating lymphoid cells via p53-independent mechanisms inhibitable by Bcl-2.. Cell.

[OCR_01321] Takahashi K., Suzuki K. (1993). Association of insulin-like growth-factor-I-induced DNA synthesis with phosphorylation and nuclear exclusion of p53 in human breast cancer MCF-7 cells.. Int J Cancer.

[OCR_01327] Whitaker S. J., Ung Y. C., McMillan T. J. (1995). DNA double-strand break induction and rejoining as determinants of human tumour cell radiosensitivity. A pulsed-field gel electrophoresis study.. Int J Radiat Biol.

[OCR_01331] Wurm R., Burnet N. G., Duggal N., Yarnold J. R., Peacock J. H. (1994). Cellular radiosensitivity and DNA damage in primary human fibroblasts.. Int J Radiat Oncol Biol Phys.

[OCR_01337] Xia F., Wang X., Wang Y. H., Tsang N. M., Yandell D. W., Kelsey K. T., Liber H. L. (1995). Altered p53 status correlates with differences in sensitivity to radiation-induced mutation and apoptosis in two closely related human lymphoblast lines.. Cancer Res.

[OCR_01154] el-Deiry W. S., Tokino T., Velculescu V. E., Levy D. B., Parsons R., Trent J. M., Lin D., Mercer W. E., Kinzler K. W., Vogelstein B. (1993). WAF1, a potential mediator of p53 tumor suppression.. Cell.

